# Host-Viral Interactions: Role of Pattern Recognition Receptors (PRRs) in Human Pneumovirus Infections

**DOI:** 10.3390/pathogens2020232

**Published:** 2013-04-03

**Authors:** Deepthi Kolli, Thangam Sudha Velayutham, Antonella Casola

**Affiliations:** 1 Department of Pediatrics, 301 University Blvd, University of Texas Medical Branch at Galveston 77555, Texas, USA; E-Mails: dekolli@utmb.edu (D.K.); thvelayu@utmb.edu (T.S.V.); 2 Microbiology and Immunology, 301 University Blvd, University of Texas Medical Branch at Galveston 77555, Texas, USA; 3 Sealy Center for Vaccine Development, 301 University Blvd, University of Texas Medical Branch at Galveston 77555, Texas, USA

**Keywords:** PRRs, RSV, hMPV, TLR, RLR, NLR, PAMP, IFN, innate immunity

## Abstract

Acute respiratory tract infection (RTI) is a leading cause of morbidity and mortality worldwide and the majority of RTIs are caused by viruses, among which respiratory syncytial virus (RSV) and the closely related human metapneumovirus (hMPV) figure prominently. Host innate immune response has been implicated in recognition, protection and immune pathological mechanisms. Host-viral interactions are generally initiated via host recognition of pathogen-associated molecular patterns (PAMPs) of the virus. This recognition occurs through host pattern recognition receptors (PRRs) which are expressed on innate immune cells such as epithelial cells, dendritic cells, macrophages and neutrophils. Multiple PRR families, including Toll-like receptors (TLRs), RIG-I-like receptors (RLRs) and NOD-like receptors (NLRs), contribute significantly to viral detection, leading to induction of cytokines, chemokines and type I interferons (IFNs), which subsequently facilitate the eradication of the virus. This review focuses on the current literature on RSV and hMPV infection and the role of PRRs in establishing/mediating the infection in both *in vitro* and *in vivo* models. A better understanding of the complex interplay between these two viruses and host PRRs might lead to efficient prophylactic and therapeutic treatments, as well as the development of adequate vaccines.

## 1. Introduction

Acute respiratory tract infections remain one of the most important causes of death in both adults and children, and are the third leading cause of death in the world (WHO. The global burden of disease http://www.who.int/healthinfo/global_burden_disease/en/). Respiratory viruses are the most frequent causative agents of these acute respiratory tract infections in humans, with significant impact of morbidity and mortality worldwide. Common Respiratory viruses include rhinoviruses and enteroviruses (*Picornaviridae*), influenza viruses (*Orthomyxoviridae*), parainfluenza, metapneumoviruses and respiratory syncytial viruses (*Paramyxoviridae*), coronaviruses (*Coronaviridae*), herpesviruses and several adenoviruses [1]. Some of the clinical symptoms associated with these respiratory viruses include the common cold, acute otitis media, laryngitis, sinusitis, pneumonia, bronchiolitis, rhinorrhea and exacerbations of asthma and chronic obstructive pulmonary disease. 

**Figure 1:**
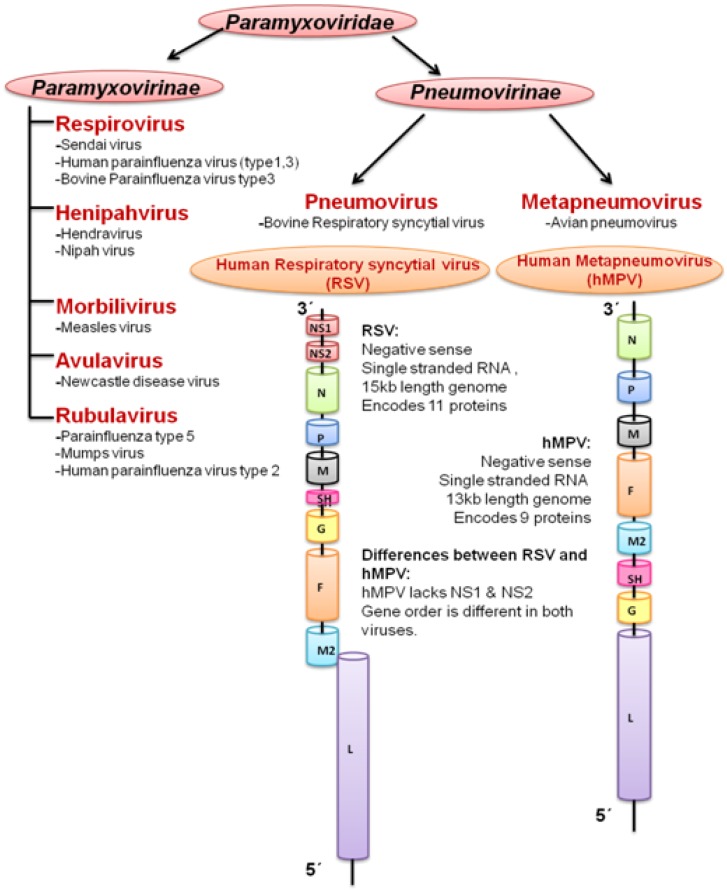
Representative members of *Paramyxoviridae* gene family and the genomic organization of respiratory syncytial virus (RSV) and human metapneumovirus (hMPV).

The *Paramyxoviridae* family belonging to the order *Mononegavirales*, includes enveloped, negative-sense, non-segmented, single-stranded RNA viruses, which are major and ubiquitous disease causing pathogens of humans and animals [2]. Among them are important viruses that cause acute respiratory morbidity, particularly in infancy, elderly and in immunocompromised subjects of any age. The family is taxonomically divided into two subfamilies, the *Paramyxovirinae*, with five genera, and the *Pneumovirinae,* which includes two genera. The classification of these viruses is based on their genome organization, morphological and biological characteristics, and sequence relationship of the encoded proteins. The pneumoviruses can be distinguished from the *Paramyxovirinae* members morphologically based on their smaller nucleocapsids [2]. In addition, pneumoviruses have differences in genome organization, the number of encoded proteins and an attachment protein that is different from that of members of the subfamily *Paramyxovirinae*. There are two genera in the *Pneumovirinae* family, the *Pneumovirus* genus that includes human and bovine respiratory syncytial virus (RSV) and the *Metapneumovirus* genus that includes human metapneumovirus (hMPV) and avian metapneumovirus (APV) (Figure 1). This review mainly focuses on the genus pneumovirus with special emphasis on RSV and hMPV. 

## 2. Respiratory Syncytial Virus (RSV)

RSV was initially isolated from the nasal secretions of young chimpanzees with sneezing and mucopurulent rhinorrhea in the year 1955 and was named as chimpanzee coryza agent (CCA) [3]. Subsequently, in the year 1956, Robert J. Chanock isolated CCA from two infants with bronchiolitis and pneumonia and based on its characteristic multinucleated giant cells within a large syncytium, it was renamed as RSV [4,5]. Since its first isolation, RSV has been identified as a leading cause of epidemic respiratory tract illness in children in the U.S. and worldwide. In fact, RSV is so ubiquitous that it will infect 100% of children before the age of 3 [6]. It is also responsible for 50% of the pneumonia cases in the first two years of life [7]. Structurally, human RSV is an enveloped virus with 10 genes distributed along 15.2 kilobases of negative-stranded RNA that encode 11 separate proteins. Eight of the RSV proteins are known to be structural and so present in the virion particle. The two non-structural proteins, NS1 and NS2, are expressed only during cell infection and are not packaged into the virion. RSV is divided into two major groups, A and B, based on the reaction of the virus with monoclonal antibodies against the major structural glycoproteins G and F [8] and by genetic analysis [9]. Each group can be further subdivided into genotypes by nucleotide sequence variability. RSV infection starts with a short course of upper respiratory symptoms such as rhinitis, however severe symptoms such as bronchiolitis and pneumonia are commonly observed in premature infants, the elderly and in immunocompromised patients [10]. 

Although the mechanism(s) underlying RSV-induced airway disease is largely unknown, experimental evidence suggests that early inflammatory and immune events of the host in response to RSV may play an important role. Following infection, RSV replicates in the respiratory mucosa leading to epithelial damage [11] and perivascular mononuclear infiltration [12]. Infected epithelial cells respond to RSV replication by producing a number of potent immunomodulatory and inflammatory mediators including cytokines [13,14,15,16] and chemokines [17,18]. 

## 3. Human Metapneumovirus (hMPV)

This virus was first characterized by Osterhaus in 2001 [19], although retrospective serological studies have revealed the existence of human metapneumovirus (hMPV) antibodies among the human population from as early as the 1950s [20]. Since its identification, hMPV has been isolated from individuals of all ages with acute respiratory tract infection worldwide [21]. Virtually, all children older than five years show 100% serologic evidence of infection [19]. Around 12% of all respiratory tract infections in children are caused by hMPV, second only to RSV [21,22,23,24]. HMPV also accounts for 10% of all hospitalizations of elderly patients with respiratory tract infections and it has been isolated from respiratory samples of a single winter season as often as parainfluenza [25]. HMPV RNA (13 kDa) encodes nine proteins that generally correspond to those of RSV, except that hMPV lacks the non-structural proteins NS1 and NS2 and the gene order is different from that of RSV (Figure 1). 

Phylogenetic analysis of strains from many countries demonstrates two distinct hMPV genotypes, A and B, which can be divided in two subgroups: A1, A2, B1 and B2 [21,22]. The clinical features associated with hMPV in children are similar to those of RSV. HMPV is associated with both upper and lower respiratory tract infections. Fever, cough, tachypnea, wheezing and hypoxia are frequently observed in infected children. A significant proportion of symptomatic children who tested positive for hMPV had co-morbidities such as a history of prematurity, chronic lung disease or complex congenital heart diseases [26]. These findings suggest that the populations of children prone to severe RSV disease may be also prone to hMPV disease. Although RSV and hMPV share similar clinic features, hMPV induces a different spectrum of immune mediators compared to RSV [27,28,29], suggesting that the host cell responses and likely the pathogenesis of lung disease are viral specific.

## 4. Pattern Recognition Receptors (PRRs)

A successful first-line of antiviral defense against respiratory viruses involves detection of the invading virus by innate immune system. This detection is mediated through recognition of pathogen associated molecular patterns (PAMPs) present on the viral pathogen, by the pattern recognition receptors (PRRs) present on host cells. PRRs are germ-line encoded proteins that are able to distinguish molecular patterns present in microorganisms but not in the host. Many of the PRR families are evolutionarily conserved. Three categories of these PRRs have been reported, including toll-like receptors (TLRs), retinoic acid-inducible gene (RIG)-I-like receptors (RLRs), and nucleotide-binding oligomerization domain (NOD)-like receptors (NLRs) [30,31,32,33]. These molecules are expressed in macrophages, lung epithelial cells, and dendritic cells as well as recruited immune cells. They have also been found in endothelial cells, stromal cells and fibroblast cells. TLRs are present on the cell membrane and in endosomes, while NLRs and RIG-I helicases are intracellular microbial sensors [33,34]. They detect several different viral components (PAMPs), such as genomic DNA, single-stranded RNA (ssRNA), double-stranded RNA (dsRNA), RNA with 5′-triphosphate ends and viral proteins. Engagement of PRRs by PAMPs leads to activation of multiple signaling pathways and a variety of transcription factors such as Nuclear Factor (NF)-κB and members of the interferon regulatory factor (IRF) family, which regulate the expression of inflammatory, immune and antiviral genes, ultimately resulting in the development of inflammation and host immunity to infections. This review mainly focuses on the current literature on the role of the various PRRs in RSV and hMPV infections both *in vitro* and *in vivo* models. A better understanding of host-pathogen interactions leading to the development of immunity and disease pathogenesis is critical to the development of efficient prophylactic/therapeutic treatments and adequate vaccines. 

## 5. Toll Like Receptors (TLRs)

Initially identified in Drosophila, TLRs are one of the largest class of PRRs and are of paramount importance for initiating and regulating immune signaling and subsequent adaptive immune responses [35]. TLRs are type I membrane glycoproteins and consist of three types of domains: extracellular ectodomains containing leucine-rich repeats (LRR), which have avidity for PAMPs; a single transmembrane domain; and an intracellular signaling domain known as Toll-interleukin (IL)-1 receptor (TIR) domain, which interacts with downstream adapter proteins. So far, 10 members of TLRs have been identified in humans, and 12 in mice. TLR 1 to 9 are conserved in both humans and mice. TLR 10 is expressed in humans but not in mice, whereas TLR 11 is expressed in mice, but not in humans. TLR 10, 12 and 13 are not well characterized and their function is only partially known [36]. These TLRs exists either as homo- or heterodimers. TLR 1, 2, 4, 5, 6, and 10 are expressed on the cell surface, whereas TLR 3, 7, 8, 9, 11, 12 and 13 [36,37,38,39] are present within endosomal compartments. Among those, TLR 3, 4, 7, 8 and 9 have been shown to be more commonly involved in the innate response to viral infections [40,41]. 

Although all TLRs share similar extracellular LRRs, they recognize very different microbial signatures. TLR 1, 2, and 6 recognize lipoproteins, TLR 3 recognizes dsRNA, TLR 4 recognizes lipopolysaccharide (LPS) and several other microbial structures and viral proteins, TLR 5 recognizes flagellin, TLR 7, 8 and 13 recognizes ssRNA, TLR 9 recognizes DNA and finally TLR 11 and 12 recognize bacterial proteins such as profilin-like proteins. Upon recognition of specific PAMPs, TLRs recruit different TIR-containing adapter molecules [*i.e.*, myeloid differentiation primary response gene 88 (MyD88), toll-interleukin 1 receptor (TIR) domain containing adaptor protein (TIRAP), TIR-domain-containing adapter-inducing interferon-β (TRIF), and thyroid hormone receptor activator molecule (TRAM)] leading to the activation of downstream signaling pathways. Two major TLR signaling pathways have been identified, *i.e.*, one that is MyD88-dependent and gives rise to strong and early activation of the transcription factor NF-κB, and a TRIF-dependent/MyD88-independent pathway that primarily drives strong activation of IRF proteins, with later activation of NF-κB. The MyD88-dependent pathway results in induction of highly NF-κB-dependent, proinflammatory genes (TNF-α, IL-1β, IL-6), while the MyD88-independent pathway leads to gene induction that is highly IRF-dependent (IFN-β, RANTES). All TLRs, with the exception of TLR 3, activate MyD88-dependent signaling pathway [42,43], whereas TLR 3 uses only MyD88 independent pathway [44,45,46]. TLR 1, 2, 4 and 6 use TIRAP as an additional adaptor to recruit MyD88 while TRAM acts as a bridge between TLR 4 and TRIF. TLR 4 is unique as it activates both MyD88 and TRIF dependent signaling and uses all four adapter molecules [45]. 

In the lung, different host cells such as epithelial cells, macrophages, dendritic cells and endothelial cells express TLRs. Lung tracheal, bronchial and alveolar epithelial cells (AECs) express TLR 1-7 and TLR 9 [47,48,49]. Recent studies by Ioannidis and colleagues showed the expression and differential distribution of TLR 1-10 in the epithelium of human trachea and suggest that this differential distribution and polarization serves tissue specific biologic needs [49]. Human and mouse alveolar macrophages were shown to express TLR 1, 2, 4, 6, 7 and -8, but not TLR 3, 5 and 9 [50,51,52]. Human myeloid DCs are equipped with TLR 1-4, 6 and 8 [53,54] whereas lung plasmacytoid DCs express high levels of TLR 7 and 9 [53,55]. Lung endothelial cells express TLR 2, 4 and 8 and may be additional TLRs [56,57] and lung fibroblasts have been shown to express TLR 2-4 and 9 [58,59,60]. The expression profile of TLRs on individual cells is modulated by infections and inflammatory mediators (e.g., tumor necrosis factor-α, interleukin-1β, *etc*.), thereby influencing the outcome of the immune responses. 

**Figure 2:**
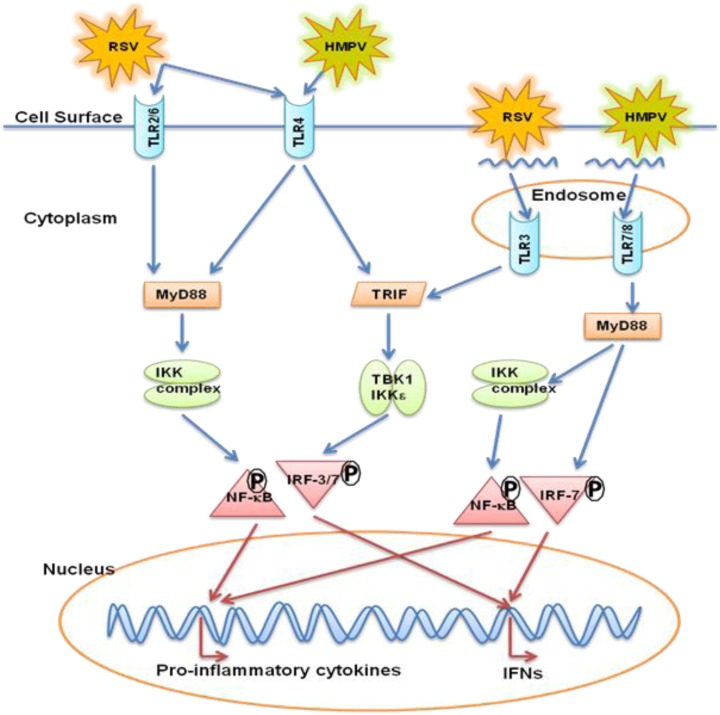
Toll-Like Receptors (TLR) signaling pathway involved in RSV and hMPV-induced gene expression. Binding to viral protein at the cell surface (TLR 2/6 and 4) or viral RNA in intracellular compartments (TLR 3 and 7/8) leads to activation of NF-κB- and IRF-dependent gene expression by engaging the adaptors (MyD88 and/or TRIF). P indicates phosphorylation.

A number of TLRs have been linked to RSV and hMPV infections, including TLRs 2-4, and TLR 7/8 [61,62,63,64,65,66]. Overall, TLR activation by these viruses seems to affect the initial phase of viral infection by modulating activation of innate immune responses. The ensuing induction of cytokines, chemokines and IFNs in the airways produces an antiviral state and modulates the adaptive immune response (see Figure 2). 

*Role of TLRs in RSV and hMPV infection.* TLR 1, 2 and 6. TLR 1, TLR 2 and TLR 6 are expressed as heterodimeric complexes (TLR 1/2; TLR 2/6) on the cell surface of immune cells and recognize a complex array of bacterial motifs (lipopeptides), as well as a diverse range of viruses (hepatitis C virus, herpes simplex virus, lymphocytic choriomeningitis virus, and human cytomegalovirus) [67,68,69,70]. Genetic analysis and RSV vaccine-based studies in mice using TLR ligands as adjuvants have indicated a possible role of TLR 2 in RSV recognition [71,72,73]. In an attempt to find a direct interaction of RSV with TLR 2, Murawski and coworkers [61] used knockout mice and provided evidence for interactions between RSV and TLR 2 and TLR 6. Their results demonstrate that TLR 2 and TLR 6, but not TLR 1, signaling can activate early innate immune responses following RSV infection. Macrophages from TLR 2 and TLR 6 knock-out (KO) mice produced lower levels of tumor necrosis factor (TNF)-α, interleukin (IL)-6, CCL2 (monocyte chemoattractant protein 1), and CCL5 (RANTES). Moreover, they reported enhanced viral replication, reduction in lung neutrophil recruitment and reduced activation of DCs at early times post-infection (p.i.), suggesting an important role for TLR 2 and TLR 6 in shaping RSV-induced innate immune response and in controlling viral burden [61]. In support to this, a critical role of TLR 2-dependent signaling in IL-1β production during RSV infection and subsequent NLR activation has been recently reported [74]. Segovia and coworkers established that RSV infection of macrophages activates TLR 2/MyD88 pathway, resulting in NF-κB activation and subsequent expression of pro-IL-1β and NLRP3 [74]. 

Even though human genetic polymorphisms in TLR 2 or 6 genes have not been indicated in RSV pathogenesis [75], a recent investigation, studying the associations and interactions of genetic polymorphisms of innate immune genes with early viral infections and susceptibility to asthma and asthma-related phenotypes, found evidence that *TLR1* (rs4543123) polymorphism interacts with RSV infection to modify the risk for atopic asthma [76]. To our knowledge, no studies have been reported so far exploring the possible involvement of TLR 2 in hMPV mediated disease. However, in a study of clinical exacerbation of hMPV-associated respiratory disease by *S. pneumonia*, increased TLR 1 expression following hMPV infection and increased TLR 2 and 6 expression in hMPV-pneumococcus coinfection, compared to single infection, has been reported [77].

TLR3. TLR 3 recognizes double-stranded (ds) RNA which constitutes the genome of some RNA viruses, and is a viral replication intermediate of ssRNA viruses such as RSV and hMPV. The localization of TLR 3 is cell-type dependent, as it is expressed on the cell surface and intracellular compartments in fibroblasts and some epithelial cells, whereas in DCs, macrophages and lung epithelial cells, it is expressed predominately in intracellular compartments [78]. Rudd *et al.* [79] were the first to demonstrate a role for TLR 3 in RSV-induced signaling production. Over expression studies showed that TLR 3 enhances RSV-induced chemokine secretion (IL-8 and RANTES) in a time- and dose-dependent manner, compared to control vector, and targeting the expression of TLR 3 with siRNA decreased the synthesis of IP-10/CXCL10 and CCL5 [79]. We and others have shown upregulation of TLR 3 mRNA and protein, as well as changes in cellular localization, in RSV-infected human airway epithelial cells [79,80]. This induction was interferon-dependent [81] and controlled phosphorylation of NF-κB Rel A at serine 276, which in turn regulates CCL5 and CXCL10 secretion [81]. 

Involvement of TLR 3 in RSV-induced lung inflammation *in vivo* has been underscored by several studies. The direct evidence for the involvement of TLR 3 in RSV-induced immunity and disease pathogenesis *in vivo* was studied for the first time by Rudd and coworkers [62] using a TLR3 KO mouse model. The TLR 3 KO mice did not show any effect on viral clearance but showed an increase in mucus production and gob5 mRNA expression, and enhanced induction of IL-13 and IL-5 [62]. Since these cytokines are linked to mucus over expression in the RSV-infected patients and asthmatics [82,83], they concluded that TLR 3 is necessary to maintain proper immune environment in the lung and avoid pathologic Th-2 biased response to RSV infection. Increased TLR 3 gene expression has also been reported in the lungs of RSV infected mice [84], as well as cotton rats [85]. Surfactant protein (SP)-C was found to contribute to alveolar defense in RSV infection by regulating TLR 3 activity, as SP-C deficient mice demonstrated delayed pulmonary clearance of RSV, increased and prolonged inflammation and increased TLR 3 gene expression and signaling in the absence of SP-C [86]. More recently it was shown that TLR 3 mediated detection of the dsRNA intermediates, generated during RSV replication cycle, is responsible for the pathogenesis and lung dysfunction [87]. 

Prior treatment with poly IC-LC (synthetic ds-RNA) leads to a dose dependent inhibition of RSV replication in mice [88], as well as in cotton rats [85], suggesting a potential role of TLR 3 agonists as therapeutic agents for RSV infection. Human studies have not found any association between TLR 3 polymorphisms and RSV-associated disease pathology. The expression levels of TLR 3 were also not very different between airway tracts of infants with RSV-associated bronchiolitis and uninfected infants [89]. 

The role of TLR 3 in hMPV infection is mostly unknown. We and others have recently reported an increase in TLR 3 expression in monocyte-derived dendritic cells (mo-DCs) [65] and in mice infected with hMPV [90]. However, we did not find a role for TLR 3 signaling in mo-DCs infected with hMPV [65]. Dou *et al*. also showed that treatment of mice with TLR 3 ligand inhibits hMPV replication and alleviates lung inflammation, possibly through activation of TLR 3 [90]. 

TLR4. TLR4 was the first PRR shown to play a role during RSV infections [63]. It is located on the plasma membrane and acts as homodimers, in contrast to other TLRs which forms heterodimers. It is expressed constitutively on monocytes, neutrophils, macrophages and in low levels intracellularly in pulmonary epithelial cells [91,92,93]. LPS is the first and main ligand of TLR4 and it has been shown that CD14 mediated association of MD-2, a non-membrane-spanning protein, with the TLR 4 ectodomain, is central to LPS-induced TLR 4 signaling [94,95,96]. Other than LPS, TLR 4 is also activated by other microbial structures and viruses, such as chlamydial Hsp60, pneumolysin, DnaK, Ebola virus glycoprotein, fibrinogen, fibronectin hyaluronan, surfactant protein A, HMGB1, hepatitis virus, hantavirus, HSV and RSV F protein [63,97,98,99,100,101,102,103,104,105,106]. Infection of human monocytes with either UV-inactivated RSV or RSV F protein results in increased production of IL-6, TNF-α and IL-β which is mediated via TLR 4 activation [63]. This RSV F mediated TLR 4 signaling was shown to be dependent on CD14, as inhibition of CD14 abrogated this response [63], as well as MD-2 expression [107]. Similar to RSV F protein, a study using 293 transfected cells shows that both human and bovine RSV activate human and bovine TLR 4 receptors, respectively, in a species specific manner, only in the presence of both MD2 and CD14, and induce NF-κB activation [108]. In contrast to these studies, another study employing 293 reporter cells showed that TLR 4/MD-2/CD14 complex was not involved in RSV infection and human TLR 4 activation by LPS remained unaffected in RSV-infected cells [109]. The authors claimed that, although isolated viral compounds such as purified RSV F protein, may bind TLR 4 and/or CD14, a direct interaction between intact RSV particles and the human TLR 4 receptor complex might not play a biological role in RSV pathogenesis [109]. 

Increased expression of TLR 4 mRNA, protein and increased membrane localization in airway epithelial cells, in response to RSV infection, has also been reported [110,111,112], suggesting a potential role in increased sensitivity of epithelial cells to environmental pollutants. In addition to NF-κB activation, TLR 4 has also been implicated in p38 MAPK activation, following RSV infection [113]. Marchant and coworkers showed that TLR4 ligation of virus, prior to host cell entry, was required to activate p38 MAPK, via MyD88, and to activate cellular internalization machinery. The need for a signaling receptor to activate virus internalization was suggested [113] based on clustering of TLR 4 at the site of virus-cell interaction and phosphorylation of downstream targets of p38 MAPK.

Several studies have suggested a role of TLR 4 in RSV-induced lung disease *in vivo*. Kurt-Jones *et al.* have shown that mice lacking TLR 4 fail to induce IL-6 production following RSV-F protein stimulation and exhibit impaired NK and CD14+ cell pulmonary trafficking, diminished NK cell function, impaired IL-12 induction and impaired virus clearance in response to RSV infection, compared to wild type mice [63,114]. Murawski *et al.* [61] also showed a decrease in cytokine response in TLR 4 KO mice, upon RSV infection, compared to wild type mice. In contrast, another study using different mouse strains lacking either both TLR4 and IL-12R or only TLR 4, found that a delay in viral clearance could be attributable to IL-12R, but not TLR 4 deficiency [115]. Such discrepancies in different studies could be attributable to differences in baseline and induced TLR expression in different mouse strains, different doses of RSV used for infection, or status of RSV stocks that present substantial changes in infectivity over time, that could highly affect the study outcomes. Work conducted in our laboratory demonstrated that RSV infection activated NF-κB in mouse lung, which was independent of viral replication but dependent on the presence of alveolar macrophages (AM) and TLR 4 expression, although the airway inflammatory response was mostly TLR 4-independent [116]. Elegant studies conducted by Shirey Ann and coworkers suggested a role of TLR 4 in resolution of RSV-induced lung inflammation [117]. They showed that RSV infection leads to generation of alternatively activated macrophages (AA-Mφ), which are important for resolution of RSV-induced lung injury, and this AA-Mφ generation was TLR 4-dependent [117]. In addition to these beneficial findings of TLR 4 involvement in RSV infection, Kunzelmann *et al.* implicated TLR 4 in causing severe RSV disease [118]. They found that TLR4 in RSV-infected mice was involved in the inhibition of sodium transport in murine epithelium causing fluid accumulation in the respiratory tract which resulted in airway congestion, increased mucus production and enhanced disease [118]. 

Immunization studies using protollin-RSV vaccine showed a significant reduction in antibody formation in TLR 4 KO mice [71,119]. TLR4 was shown to be crucial to elicit antigen-specific systemic and mucosal antibodies, while antigen-specific Th1 responses required mainly MyD88 signaling. Similarly, in the cotton rat model, inclusion of monophosphoryl lipid A (MPL), a TLR 4 ligand, in the FI-RSV formulation was reported to mitigate the lung pathology associated with vaccine-enhanced disease by a dramatic reduction in levels of Th1- and Th2-type cytokines and chemokines normally elicited in response to RSV challenge [120]. Phosphatidylglycerol (PG), an antagonist of LPS binding protein (LBP), and CD14 has been used as a therapeutic agent in both *in vitro* and *in vivo* studies. Treatment of bronchial epithelial cells with pulmonary surfactant phospholipid, palmitoyl-oleoyl-phosphatidylglycerol (POPG) significantly inhibited interleukin-6 and -8 productions, as well as the cytopathic effect induced by RSV infection. Further, administration of POPG to mice, together with RSV infection, almost completely eliminated the recovery of virus from the lungs at 3 and 5 days p.i., abrogated IFN-γ production and the enhanced expression of surfactant protein D (SP-D) [121]. 

Gagro and coworkers assessed for the first time the expression of TLR 4 in RSV infected patients and found that RSV infection induces TLR 4 expression on monocytes during the acute phase of RSV bronchiolitis [122]. This increase in TLR 4 expression was associated with reduced oxygen saturation, suggesting that TLR 4 expression was related to the severity of illness in RSV-infected infants.

A different study associated low TLR 4 expression in neutrophils from blood as well as BAL to the severity of RSV bronchiolitis [123]. Two human TLR 4 gene polymorphisms, A896G and C1196T, encoding amino acid changes Asp299Gly and Thr399Ile, within the extracellular domain of TLR 4, leading to disruption of LPS signaling [124], have been associated with an increased risk of severe RSV bronchiolitis in infants and young Caucasian children [75,125,126,127,128,129]. Similar studies in different populations, however, failed to show an association between these two polymorphisms and severity of RSV disease [130,131,132,133]. This could be explained on the basis that susceptibility to severe RSV infection and the association studies are influenced by several other factors like environment, allelic variation in TLR 4, which may widen the repertoire of host responses in different RSV epidemic, age of patients; selection of controls, and ethnic background. 

Regarding the role of TLR 4 in hMPV infection, recent studies conducted in our laboratory have shown that TLR 4 plays an important role in hMPV induced innate immune responses [65]. Bone marrow-derived dendritic cells (BMDCs) from TLR 4 deficient mice, using two different strains (C3-Tlr4Lps-d/J mice with a spontaneous point mutation in the intracellular domain of TLR 4 and C57BL/10ScNJ mice carry a deletion of the TLR 4 gene) showed a significant reduction in hMPV-induced cytokine, chemokine and type I interferon production, compared to cells isolated from wild type mice [65]. Moreover, using these TLR 4 KO mouse models of hMPV infection, we found that TLR 4 plays an important role in the regulation of hMPV-induced inflammatory responses and disease pathogenesis *in vivo* [134]. Mice lacking TLR 4 showed less clinical disease, demonstrated by reduced body weight airway obstruction and hyperresponsiveness (AHR), compared to wild type mice. When inflammatory mediators were measured in bronchoalveolar lavage fluid on different days post-infection, significantly lower levels of proinflammatory cytokines (IL-1β, IL-6 and TNF-α), immunomodulatory cytokines (GM-CSF, IL-12 p40, IL-17) and chemokines (MCP-1, MIP-1α) were detected in the TLR 4 deficient mice compared to the wild type. Accordingly, inflammatory cell recruitment in the BAL, lungs, as well as in lymph nodes, was also significantly reduced. These results indicate that TLR 4 is important for activation of the innate immune response to hMPV infection; however, it also contributes to disease pathogenesis [134].

TLR 7, 8 and 9. TLR 7, 8 and 9 are preferentially confined to intracellular compartments, such as the endoplasmic reticulum (ER), endosomes, lysosomes, and endolysosomes, rather than being expressed at the cell surface, and recognize nucleic acid motifs. TLR 7 and 8 recognizes uridine rich or uridine/guanosine rich ssRNA of both viral and host origin, while TLR 9 recognizes bacterial and viral DNA that is rich in CpG-DNA motifs. TLR 7, 8 and 9 signaling induces antiviral cytokine, chemokine and IFN-α secretion through MyD88-dependent activation and recruitment of NF-κB and IRF-7. Since RSV is a single stranded negative sense RNA virus, both dsRNA and ssRNA species are formed, which provide targets for recognition by TLR 3 and TLR 7/8, respectively [135]. Despite the fact that expression of these TLRs in lung epithelial cells (main target for RSV infection) is very low, a role of these TLRs in RSV infection has been suggested. Studies conducted by Lindemans and coworkers have shown the involvement of endosomal TLRs in RSV infection [136]. They showed that RSV infection enhanced granulocyte life span, by inhibiting their apoptosis, and this delay in apoptosis was likely mediated by TLR 7/8, based on its requirement for endosomal internalization. Using hypereosinophilic transgenic mice, another group demonstrated that eosinophils express TLR 7 and secrete antiviral genes such as IFN-β and (nitric oxide synthase (NOS) 2 in a TLR **7**-MyD88-IRF-7-dependent manner in response to RSV infection [137]. Increased TLR 7 expression in mouse lung has also been reported, although with different kinetics following RSV infection [64,84]. TLR 7 deficiency mice showed increased pathologic responses following RSV infection, especially the production of mucus, increased number of airway goblet cells, enhanced induction of IL-17, as well as Th2 cytokines IL-4 and IL-13 [64]. The increased pathology was attributed to the skewing of the dendritic cell responses away from Th1 promoting cytokines (IL-12) and favoring Th17-promoting cytokines (IL-23) in the lungs of TLR 7 deficient mice [64]. More recently, Mc Gill and coworkers reported a previously unrecognized ability of bovine neonatal γδ T cells to respond to stimulation via TLR 3 or TLR 7 and indicate their contribution to the recruitment of inflammatory populations during RSV infection [138]. 

In addition to the *in vitro* and mouse studies, changes in TLR 7 and 8 expressions has been reported in blood cells isolated from infants infected with RSV. One study showed enhanced TLR 7/8 in PBMCs of infected infants [89], while another reported a significantly reduced TLR 8 levels on monocytes during acute RSV infection, compared to healthy infants [139], which could compromise virus recognition by monocytes/macrophage and lead to less efficient anti-RSV immune response and the development of severe disease.

Johnson and colleagues evaluated the impact of TLR 7/8 and TLR 9 activation on RSV disease by administering TLR 7/8 and TLR 9 agonists during primary or formaline inactivated (F1)-RSV immunization [140]. Even though a reduction in Th2 responses in vaccine-enhanced disease was observed using TLR 9 agonists, all TLR agonists used increased clinical symptoms and pulmonary inflammation in primary RSV infection [140]. Tayyari *et al.* [141] evaluated the immunotherapy capability of TLR 9 agonists, CpG oligodeoxynucleotides (ODN) on ovalbumin sensitization of guinea pigs with and without RSV infection. The authors showed that in RSV-infected, ova-sensitized animals, CpG ODN caused significant reduction of airway T cells, eosinophils, increased lung IFN-γ/IL-5 ratio and decreased OVA-specific IgG_1_ antibodies compared to uninfected, ova-sensitized animals. Moreover, their results showed that CpG ODN treatment protected guinea pigs against RSV infection and this was attributed to the induction of type I IFN by CpG ODN-stimulated pDCs during RSV infection. In a different study, a combined treatment of IL-4 and fractalkine antagonist with CpG ODN completely prevented RSV replication in ciliary epithelial cells and the skewing of the Th1/Th2 balance toward Th2 cytokines [142]. Finally, exposure to CpG ODN, prior to neonatal RSV infection in mice, is protective against enhanced disease during secondary adult RSV challenge, with a reduction in viral load and an increase in Th1 responses [143].

**Figure 3:**
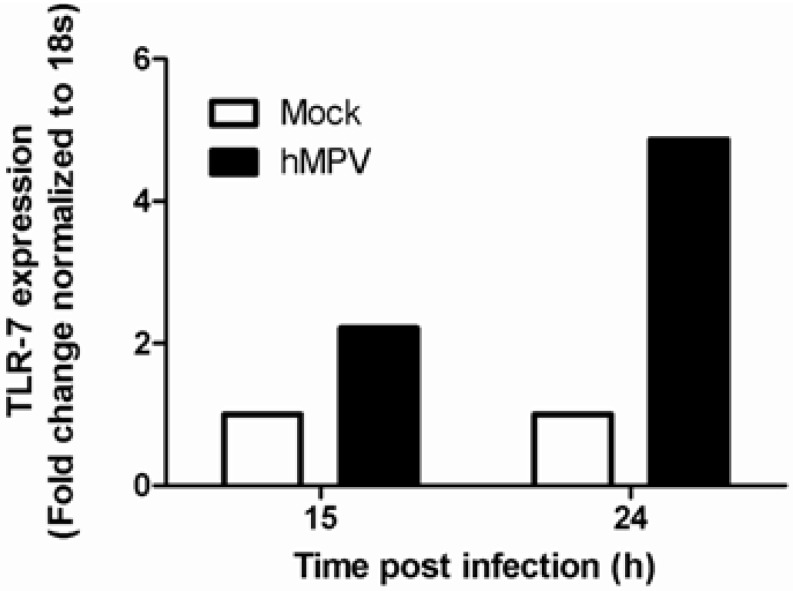
Upregulation of TLR 7 following hMPV infection in monocyte derived dendritic cells. MoDCs were infected with hMPV (MOI 3) and cells were harvested at different time’s p.i. to prepare total RNA for analysis of TLR 7. Results are representative of two separate experiments.

Regarding hMPV infection, TLR 7 has also been shown to play a role in viral recognition and induction of type I IFN in response to infection. TLR 7-deficient, but not RIG-I deficient pDC showed reduced IFN-β secretion following hMPV infection, compared to wild type cells [66]. We have also observed a time dependent increase in TLR 7 expression in viral-infected mo-DCs **(**Figure 3**)**, although it did not seem to play a role in hMPV-induced signaling [65]. In an *in vivo* model of infection, hMPV has been shown to up-regulate the expression of many TLRs, including TLR 7, in the lungs of BALB/c mice and it was suggested that the TLR 7/8 pathway might play an important role in the initiation of innate immune responses [90]. In a murine model of clinical exacerbation of hMPV-associated respiratory disease by *S. pneumoniae*, increased TLR 9 expression by hMPV infection and increased TLR 7 expression, following hMPV-pneumococcus coinfection, has been reported [77]. Table 1 summarizes the different studies addressing the involvement of different PRRS in RSV and hMPV infections.

**Table 1:** Comparison of studies addressing the involvement of different pattern recognition receptors (PRRS) in RSV and hMPV infections.

Pattern Recognition Receptors	Virus	Studies	
		*In vivo* / *ex vivo* (Ref)	*In vitro* (Ref)
**TLR 1/2,6**	RSV	61, 71–74	74
	hMPV	-	-
**TLR 3**	RSV	62, 84–88	79–81
	hMPV	65, 90	-
**TLR 4**	RSV	61, 63, 114–123	107–113, 118, 121
	hMPV	65, 134	-
**TLR 7/ 8**	RSV	64, 89, 136–140	-
	hMPV	66, 65, 77, 90	66
**TLR 9**	RSV	140, 141, 143, 200	-
	hMPV	77	-
**MyD88**	RSV	166	-
	hMPV	-	-
**RIG-I**	RSV	166–169	33, 81, 164, 165, 168, 170–172
	hMPV	66	66, 162, 174, 176
**MDA5**	RSV	-	172
	hMPV	173	-
**MAVS**	RSV	166, 167	176
	hMPV	-	175, 176
**NLRP3**	RSV	74	74
	hMPV	-	-
**NOD2**	RSV	32, 169, 200	32
	hMPV	-	-

## 6. Retinoic Acid Inducible Gene like Receptors (RLRs)

RLRs, which belong to superfamily 2 (SF2) helicases/ATPases, are a family of intracellular PRRs involved in the recognition of cytosolic pathogenic-derived RNA in most cells. Initially identified by transcriptional profiling, the RLR family includes three members, retinoic acid-inducible gene I (RIG-I), also known as DDX58 [144], melanoma differentiation-associated gene 5 (MDA-5), also known as helicard or IFIH1 [145], and laboratory of genetics and physiology 2 (LGP2) [146,147]. RLRs have the capacity to distinguish between self and non-self RNA in the cytoplasm and initiate host defenses by triggering innate immune signaling cascades against invading viruses [148]. Structurally, RIG-I and MDA-5 are composed of three major domains, a C-terminal regulatory/repressor domain (RD) domain) involved in specific pattern recognition, a central DEAD box helicase/ATPase domain that interacts with dsRNAs, which in turn induces their ATP catalytic activity, and two N-terminal caspase activation and recruitment domains (CARDs), responsible for activating downstream signaling pathways. LGP2 lacks the CARD domains and does not directly sense viral RNA [145,148,149], and it has been suggested to function as a negative regulator of RIG-I/MDA-5-dependent signaling [147,148,150]. RIG-I senses preferentially the nascent 5’ triphosphate moiety of viral genomes or virus derived transcripts of negative-sense ssRNA viruses, whereas MDA5 is activated by long dsRNA, a typical intermediate of the replication of plus-sense ssRNA viruses. Upon activation by specific RNA features, RIG-I or MDA5 associate with the CARD containing adaptor protein, mitochondrial antiviral signaling (MAVS), also known as IFN-β promoter stimulator 1 (IPS1), virus-induced signaling adapter (VISA), and CARD adapter inducing IFN-β (CARDIF) [151,152,153,154], which is located in the outer mitochondrial membrane, as well as in peroxisomes [151,152,153,154]. The interaction of RIG-I or MDA-5 with MAVS leads to dimer formation [155,156], and subsequent activation of the serine/threonine kinases IKKα/β and IKKε/TBK-1. IKKα/β leads to NF-κB activation, while IKKε/TBK-1 phosphorylates IRF-3 and IRF-7, triggering the expression of type I IFN [157,158,159].

**Figure 4:**
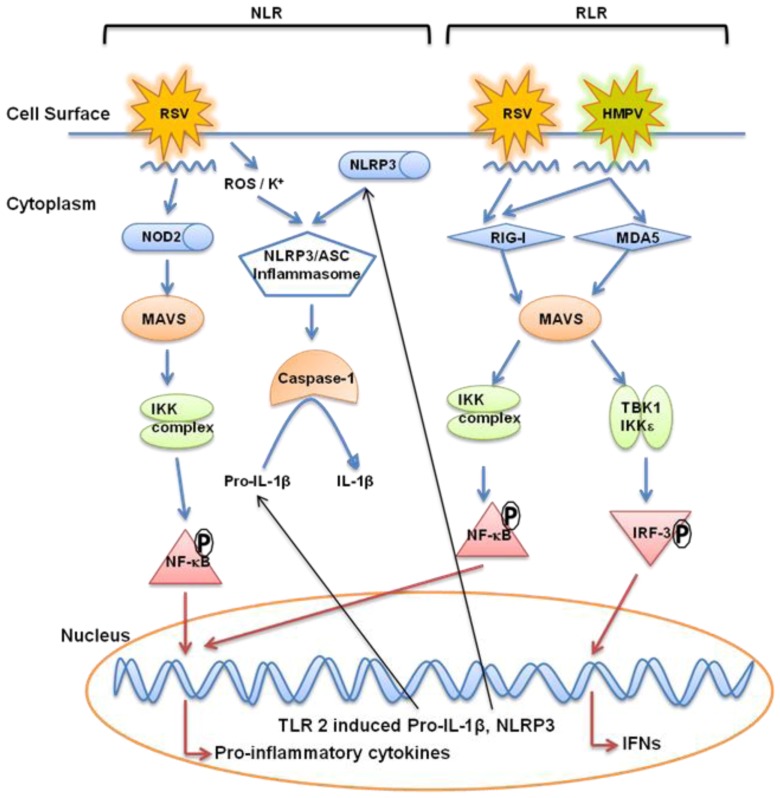
Intracellular PRR signaling pathways involved in RSV and hMPV-induced gene expression. Production of specific RNA moieties during viral replication leads to activation of the either RLR or NLR pathway. Binding to the adapter molecule (MAVS) activates NF-κB and/or IRF 3 leading to proinflammatory/immune gene expression. P indicates phosphorylation.

RLRs play an important role in the production of type I IFNs, as well as cytokines and chemokines, in most cell types, such as fibroblasts, epithelial cells, macrophages and conventional dendritic cells (cDCs), with the exception of pDCs, which produce IFNs in the absence of RLR signaling [160]. The role of RLRs as specific PRRs has been established in several viral infections [33,161]. RIG-I is particularly important in several negative-sense RNA viruses, such as Newcastle disease virus, vesicular stomatitis virus, influenza A virus, Sendai virus, RSV and hMPV [33,81,160,161,162] where as MDA-5 plays a major role in some positive-sense RNA viruses, such as picornaviruses, poliovirus and encephalomyocarditis virus (EMCV) [161,163]. Figure 4 depicts the involvement of RLR and NLR signaling pathway in RSV and hMPV infection.

*RLRs in RSV and hMPV infection.* The involvement of CARD-helicase pattern-recognition receptors (RIG-I/MDA-5) in RSV infection was first reported by Sasai and coworkers in 2006 [164]. Their study demonstrated that RIG-I is a key molecule in RSV-mediated, replication-dependent IFN-β induction in HeLa cells. This function was mediated through NAK-associated protein 1 (NAP1), the regulatory subunit of the kinase complex IKKε and TBK1. We have also showed a fundamental role of RIG-I in RSV-induced signaling in airway epithelial cells [81]. Our results showed RSV infection induced RIG-I and MDA-5 expression and that RIG-I, but not MDA-5, specifically binds to RSV RNA. Using siRNA-mediated RIG-I knockdown approach, we demonstrated that RIG-I was necessary for nuclear translocation of both NF-κB and IRF-3 in response to RSV infection [81]. RIG-I was also shown to be involved in NF-κB activation, in response to RSV infection, through a “cross-talk” pathway involving the noncanonical NIK.IKKα complex [165]. In a study investigating how viruses from distinct genera initiate innate immune response, it was shown that expression of IRF-3-dependent genes, including ISG15, ISG54, and ISG56, in mouse embryonic fibroblasts following SV, NDV and RSV infection was dependent on RIG-I expression [33]. Cells lacking RIG-I were overall more permissive to RSV and NDV infection, indicating that RIG-I actions restrict initial infection. In addition, these studies suggested that MDA-5 might play an auxiliary role in amplifying innate immune signaling initiated by RIG-I during paramyxovirus infection [33] (refer Table 1). 

In addition to epithelial cells and fibroblasts, the involvement of RLR pathway in RSV infection has been demonstrated in antigen presenting cells (APCs), such as macrophages and dendritic cells [166,167]. BMDMs and dendritic cells obtained from mouse deficient in MAVS showed significant reduction in the production of type I interferon following RSV infection. Similar results have been obtained in an *in vivo* model of infection, where MAVS deficient mice produced reduced amount of type I IFN in response to RSV infection. Secretion of other proinflammatory cytokines, including IL-6, TNF-α, MCP-1, and IL-1β, was also depended on MAVS for their expression [167]. Furthermore, an important role of MAVS in the generation of anti-RSV antibodies during the adaptive phase of the antiviral response has been reported. Work by Lukacs group reported enhanced lung inflammation, defective viral clearance at 8 days p.i., increased T cell activation and Th1 phenotype in MAVS deficient mice, compared to wild type [167]. Using bone marrow chimeras, they proposed a differential regulation of inflammation and viral clearance by IPS-1 expression in immune and nonimmune cell populations. 

RSV is the most frequently detected virus in infants with bronchiolitis and it has been shown that infants with RSV bronchiolitis have relatively high levels of the gene expression of several PRRs, especially RIG-I, in their airway tract, compared to infants who have bronchiolitis without a respiratory virus infection. Moreover, a positive correlation between RIG-1 mRNA levels and the viral load of RSV was observed in these RSV-infected infants [89]. Using human catalytic subunit of telomerase reverse transcriptase-transfected human primary nasal epithelial cells (hTERT-NECs), Okabayashi and coworkers reported that type III interferon, not type I, was the predominant IFN induced by RSV in nasal epithelial cells and it was RIG-I-dependent [168]. More recently, increased RIG-I expression following RSV infection of human PBMCs isolated from healthy volunteers has also been reported [169].

The importance of RIG-I in RSV recognition has also been suggested by RSV protein mediated antagonism of IFN production, which is beyond the scope of this review. RSV NS2 protein inhibits RIG-I-dependent IFN promoter activation by binding to the N-terminal CARD domains of RIG-I, thereby inhibiting its interaction with MAVS [170], whereas NS1 protein binds to MAVS, also interfering with RIG-I-MAVS interaction and subsequent signaling, without significantly affecting expression of either RIG-I or MAVS [171]. Similarly, RSV nucleoprotein has been shown to colocalize with RIG-I and MDA-5 in viral inclusion bodies, and to antagonize the innate immune responses by interacting with MAVS [172]. 

Similar to RSV infection, we have reported, for the first time, the role of RIG-I in hMPV-induced cellular signaling [162]. In airway epithelial cells, hMPV induces both RIG-I and MDA-5 expression, and RIG-I, but not MDA-5, plays a fundamental role in hMPV-induced cellular signaling, as inhibition of RIG-I expression significantly decreased activation of IRF and NF-*κ*B transcription factors and production of type I IFN and proinflammatory cytokines and chemokines [162]. RIG-I-dependent signaling was also necessary to induce a cellular antiviral state, as reduction of RIG-I expression resulted in enhanced HMPV replication [162]. Similar results have been observed in additional cell lines, as well as primary human cells, such as monocytes, and 5′ triphosphate RNA was identified as the hMPV ligand for triggering RIG-I dependent IFN-β response [66]. 

Recently, the importance of MDA-5 in hMPV infection in dendritic cells (human and mouse), as well as in an experimental mouse model, has been reported [173]. In human myeloid dendritic cells, as well as BMDCs, hMPV-induced IFN-α/β expression, as well as IRF-3 and IRF-7 activation, was dependent on MDA-5. Mice lacking MDA-5 exhibited impaired antiviral responses (decreased Type I IFN, increased viral replication), increased disease severity (prolonged body weight loss) and exacerbated pulmonary inflammation (increased cellular infiltration and cytokines, chemokines) following hMPV infection, compared to wild type mice [173].

In addition to these studies, we and others have also shown the importance of RIG-I in hMPV-mediated immune evasion. G protein from hMPV A2 strain has been shown to associate with RIG-I and inhibit RIG-I-dependent gene transcription, but not MDA-5 and MAVS [174], whereas M2-2 protein antagonizes MAVS-dependent antiviral responses [175]. Phosphoprotein of hMPV B1 has also been implicated in preventing RIG-I from sensing the viral RNA [66]. Using cell culture based assays, we have shown that hMPV G protein inhibits RIG-I by blocking its association with MAVS and the formation of the mitochondrial signalsome, leading to impaired IRF and NF-κB activation [176]. 

## 7. Nucleotide Binding Oligomerization Domain-Like Receptor (NLRs)

The NLR family of pattern recognition molecules, also called Nod-leucine-rich repeats (NOD-LRRs) [177], NACHT-LRRs (NAIP, CIITA, HER-E, TP-1, leucine-rich repeat) [178], or CATEPILLER proteins (CARD, transcription enhancer, purine binding, pyrin, lots of leucine repeats) [179,180,181], are specialized intracellular cytoplasmic sensors that are involved in a diverse array of processes required for host immune responses against invading pathogens. They belong to the signal transduction ATPases with numerous domains (STAND) subclass of the AAA-ATPase superfamily. In humans, the NLR family is composed of 22 proteins, and at least 33 NLR genes have been identified in mice [182]. The NLR family of PRRs is characterized by their tripartite domain architecture that contains a variable C-terminal leucine-rich repeat (LRR) that detects PAMPs, a central nucleotide-binding oligomerization (NOD) domain, which mediates nucleotide binding, ATPase activity and self-oligomerization, which occurs during activation, and an N-terminal protein-protein interaction domain that recruits downstream effector molecules. There are four possible N-terminal domains: pyrin domain (PYD) (NLRP, a.k.a. PAN, NALP, or PYPAF), caspase recruitment domain (NLRC, a.k.a. NOD), baculovirus inhibitor repeat (BIR) (NAIP), and acidic transactivator domain [182,183]. The well-characterized members of the NLR family include NOD1, NOD2, NIAP, NLRP1, NLRP3 (NOD-like receptor family, pryin domain containing 3; also known as NALP3 and cryopyrin), and NLRC4. NOD1 and NOD2 recognize intracellular bacterial cell products, and NALP3 responds to multiple stimuli including bacterial and viral RNA and DNA, small antiviral compounds *etc* [180,182]. 

Similar to TLRs and RLRs, recognition of their specific PAMP leads to stimulation of the intracellular NLRs and subsequent activation of downstream signaling pathways for the production of proinflammatory mediators to defend the host against infection. However, the end targets of NLR signaling are not the same for all NLRs. The three major activation targets of NLR signaling after PAMP recognition are NF-κB and MAPKs (NOD1 and NOD 2) [46,184,185], and caspase-1 (NLRP1, NLRP3, NLRC4) [186,187,188,189]. Some NLR members instead of promoting NF-κB activation may also have a negative regulatory role (NLRP2, NLRP12) [190,191,192]. A number of NLR family members (NLRP1, NLRP3, NLRC4) can form multiprotein complexes, called inflammasomes, and are capable of activating the cysteine protease caspase-1 in response to a wide range of stimuli including both microbesl and self-molecules. These NLRs induce the recruitment of the adaptor molecule ASC (apoptosis associated speck-like protein containing a CARD), leading to the processing and activation of pro-IL-1β and IL-18 through caspase-1 [186,189,193,194]. Although NLRs have been shown to be primarily expressed in immune cells, including monocytes, lymphocytes and antigen-presenting cells (APCs) such as macrophages and dendritic cells, they can also be expressed in nonimmune cells, including epithelial and mesothelial cells [182,195,196]. 

As mentioned above, the NLRP3 inflammasome is activated by multiple stimuli including a variety of viruses, suggesting a common pathway for viral detection by host cells [74,189,197]. The first evidence for the involvement of NLR-containing inflammasomes in viral infection came from a study in which Sendai virus and influenza A virus were shown to stimulate caspase-1 activation and the production of IL-1β and IL-18 [189]. Subsequent studies have shown that influenza A virus can activate NLRP3 in various cell types *in vitro*, including mouse BMDCs and macrophages, human nasal airway epithelial cells and the human monocyte cell line THP-1 [197,198]. Many viruses, including RSV, activate caspase-1 and induce IL-1β and IL-18 production [199] and a critical role of NLRP3/ASC inflammasome activation for RSV induced IL-1β production has been reported [74]. Segovia and coworkers [74] demonstrated that RSV infection in mouse bone marrow derived macrophages induces TLR2/MyD88 pathway, leading to activation of NF-κB, which in turn translocates to the nucleus to transactivate pro-IL-1β and NLRP3 genes. Reactive oxygen species and potassium efflux (via stimulation of ATP-sensitive potassium channels) generated in infected cells trigger formation of NLRP3/ASC inflammasome complex, which cleaves pro-caspase-1 to generate active caspase-1 which ultimately leads to the secretion of IL-1β. However, a direct interaction between NLRP3 and RSV RNA has not been demonstrated yet, and the precise molecular mechanism of NLRP3 signaling is not well known (see Figure 4). 

Involvement of another NLR family of PRR, NOD2, in cellular signaling elicited by several RNA viruses, including VSV, RSV, parainfluenza virus 3, and influenza A, has been recently reported [32]. Studies conducted by Sabbah and coworkers have indicated for the first time that NOD2 can recognize ssRNA virus and is involved in innate antiviral responses in human bronchial epithelial cells, macrophages, and embryonic fibroblasts. Both synthetic ssRNA and ssRNA viral genomes activated IRF-3 in a NOD2- and MAVS-dependent manner, and infection with RSV resulted in increased NOD2 expression, leading to IFN production within 2 h p.i., whereas other PRRs (e.g., RIG-I) activate the IRF-3-IFN pathway during a later infection period. NOD2 was shown to translocate to the mitochondria and to interact with MAVS to induce activation of both IRF-3 and NF-κB. The importance of NOD2 in antiviral defenses was shown by the increased body weight loss, decreased type I IFN production and increased proinflammatory cytokine and chemokine production, enhanced lung disease and virus susceptibility of NOD2-deficient mice infected with RSV, compared to wild type [32]. Similarly, macrophages and mice that lacked NOD2 had decreased IRF-3 phosphorylation and production of type I IFNs in response to influenza A and parainfluenza viruses and *NOD2*^−/−^ cells were deficient in their ability to inhibit VSV replication. Recently, Vissers and coworkers stimulated human PBMCs with RSV and the common bacterial ligand MDP, and showed that primary infection with RSV induces IFN-β, which leads to the upregulation of NOD2 and subsequent signaling of NOD2 by MDP then induces a higher proinflammatory cytokine response [169]. More recently, the potential use of NOD2 ligands in combination with TLR 9 ligands as adjuvant in inducing RSV-specific immunity has been tested [200]. Non-replicating RSV antigen usually does not induce a strong mucosal immune response and mucosal administration does not seem to prime for enhanced disease. Shafique and coworkers showed that beta-propiolactone (BPL)-inactivated RSV (BPL-RSV), supplemented with CpG ODN (TLR9 ligand) and L18-MDP (NOD2 Ligand), induced stronger activation of APC *in vitro*, and induction of local IgA responses in the respiratory tract after immunization *in vivo*. Their results indicate that addition of TLR 9/NOD2 ligands to inactivated RSV promoted affinity maturation of RSV-specific IgG antibodies, Th1-skewed response and significantly improved the protection efficacy against a challenge with infectious virus, without inducing enhanced disease, suggesting that mucosal immunization with inactivated RSV antigen supplemented with TLR9/NOD2 ligands is a promising approach to induce RSV-specific immunity. To our knowledge, no studies have been published so far dissecting the involvement of NLRs in hMPV infection (refer to Table 1). 

In summary, Virus-induced respiratory disease accounts for the majority of hospitalizations of infants and young children and the major viral causes of lower respiratory tract disease are RSV and hMPV in addition to rhinovirus, parainfluenza virus 3 (PIV-3), and influenza. Important progress has been made in the last decade delineating the critical role of several PRRs in RSV and hMPV infections, in terms of recognition of viral proteins and/or RNA by host and subsequent initiation and orchestration of host immune responses. Even though TLRs play a distinct role in mediating RSV/hMPV infections, the role of other PRRs such as RLRs and NLRs is gaining importance. A better understanding of how host recognizes and differentiates these two viruses and mediates cellular signaling and innate and adaptive immune responses is crucial for improving therapeutic approaches and the development of better vaccines against these two important viral pathogens.
